# From Aeromedical Evacuation to Planetary Habitation: A PubMed‐Based Historical Mapping Review of Aerospace Nursing (1946–2025)

**DOI:** 10.1111/nin.70098

**Published:** 2026-03-30

**Authors:** Kazumi Kubota, Satoko Tsuda, Anna Kubota

**Affiliations:** ^1^ Research Organization Shimonoseki City University Shimonoseki Japan; ^2^ Department of Healthcare Information Management The University of Tokyo Hospital Tokyo Japan; ^3^ Health and Global Policy Institute Tokyo Japan; ^4^ College of Life and Health Sciences Chubu University Kasugai Aichi Japan; ^5^ Department of Health Policy and Management Keio University School of Medicine Tokyo Japan

**Keywords:** aeromedical evacuation, aerospace nursing, commercial spaceflight, historical mapping review, microgravity, nursing theory, space nursing, workforce development

## Abstract

Civilian spaceflight and long‐duration missions are expanding, creating a need for nursing frameworks that extend beyond survival‐focused transport care toward habitation‐oriented support. This PubMed‐based historical mapping review traced the evolution of aerospace nursing across 1946–2025 and examined how clinical priorities, target populations, and theoretical lenses have shifted over time. A focused PubMed search retrieved 208 records (last searched December 30, 2025). Title and abstract screening excluded clearly non‐relevant records (*n* = 12); no full‐text records were excluded. The remaining 196 records were synthesized using thematic and chronological mapping and summarized in an evolutionary matrix. Four phases were identified: Origins (1946–1970s), centered on aeromedical evacuation and survival during transport; Professionalization (1980s–1990s), emphasizing provider well‐being and the emergence of nursing theory in space contexts; Clinical Adaptation (2000s–2010s), focusing on procedural adaptation, training, and safety practices in altered gravity; and the New Frontier (2020s–2025), addressing civilian participation and competencies for long‐duration missions. Across eight decades, the field shifted from guarding survival to facilitating habitation. Persistent gaps include everyday living support, life‐course and reproductive care, and chronic disease management in altered gravity, with implications for workforce development and policy.

## 1 Introduction

**Figure 1:**
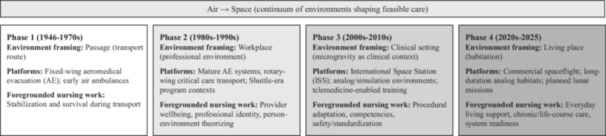
Conceptual scope and dominant framings of aerospace nursing across four phases. The diagram summarizes how PubMed‐indexed literature (1946–2025) has tended to frame the aerospace environment—as a passage, workplace, clinical setting, and living place—and how each framing foregrounds different mission‐critical nursing work across air and space platforms.

The year 2021 marked a definitive turning point in human history with the successful launch of the first all‐civilian spaceflight mission, signaling the dawn of the “Space Tourism Era.” Space is no longer the exclusive domain of carefully selected, elite professional astronauts; it is rapidly transitioning into a sphere of activity for diverse populations, including older adults, individuals with physical disabilities, and those with pre‐existing chronic health conditions. As humanity extends its footprint from low Earth orbit to the Moon via the Artemis program, and eventually to Mars, the focus of health support in aerospace environments faces a critical paradigm shift: moving from ensuring mere “survival” in extreme environments to supporting long‐term “habitation” and everyday living. Research on complex spaceflight operations further underscores that mission timelines, distributed teams, and constrained resources will shape what care is feasible and how it is prioritized, making sustained health support a mission‐relevant systems problem rather than an emergency‐only contingency (Marquez et al. 2023). In this manuscript, we use “aerospace” to refer to health and care contexts spanning air and space environments—settings in which the physical environment and operational constraints, including noise, vibration, altered gravity, limited privacy, and delayed evacuation, meaningfully shape what care is feasible. We use “aerospace nursing” to refer to nursing practice, education, and knowledge development concerned with providing care in, for, or because of these environments, ranging from time‐critical stabilization during transport to the longer horizon work of sustaining everyday functioning, comfort, and dignity in off‐Earth living conditions. Beyond tourism, broader conversations about planetary health, environmental limits, and long‐term human resilience also increasingly shape the rationale for sustained human presence beyond Earth, reinforcing the importance of nursing frameworks that address “living” and not only surviving. We use “habitation” primarily as a conceptual and prospective frame for nursing knowledge needs emerging from longer duration missions and civilian participation. Our synthesis maps what is visible in the PubMed‐indexed literature through 2025 and should be read as a map of PubMed‐indexed discourse rather than as evidence of established clinical practice in off‐Earth colonies (Figure 1).

In this evolving landscape, the discipline of nursing—fundamentally centered on holistic care and adaptation to the environment—is poised to play a pivotal role. Historically, aerospace nursing emerged from World War II aeromedical evacuation, where flight nurses developed practices for caring for wounded soldiers during transport (Barger 1979). Early accounts described nursing work in air ambulances and evacuation chains, focusing on oxygen needs, monitoring, and stabilization in noisy, vibrating cabins (Graham 1951; Lazaro 1949; Purvis 1947; Thorp 1949). As aviation medicine matured, publications also articulated problems in aero medical nursing and practical innovations (e.g., equipment kits) intended to support care under flight constraints (Thorp 1949; Thorp and Stein 1948).

Over time, aerospace nursing also began to engage with broader physiological questions relevant to altered gravity and extreme environments. Laboratory work on altered gravity illustrates that gravity acts as a continuum influencing biological function, reinforcing that “habitation” involves more than episodic emergencies (Plaut et al. 2003). In parallel, theoretical writings positioned space nursing as a legitimate scientific endeavor and foregrounded person–environment relations as central to nursing knowledge (Malinski 1990; Rogers 1990). Professional discussions further framed space nursing as a frontier and “professional challenge,” emphasizing that nursing's conceptual resources must extend beyond transport survival toward holistic adaptation (Barrett 1991; Perrin 1985). Nursing contributions to space programs also became more visible, illustrating that nursing work intersects with mission systems, preparation, and research—not only bedside tasks (Czerwinski et al. 2000).

However, despite this rich history spanning nearly 80 years, the evolution of aerospace nursing remains fragmented within the literature. While biomedical data exist regarding physiological changes in professional astronauts, nursing implications for civilians living in space remain less clearly articulated. Evidence is sparse in domains central to habitation, such as hygiene, elimination, sleep comfort, privacy, mental health support for isolation, and chronic disease management in altered gravity. Recent work has begun to emphasize competencies for disaster situations, highlighting that civilian spaceflight will require preparedness for low‐probability, high‐consequence events (Paula et al. 2024). Likewise, organ‐focused nursing perspectives (e.g., respiratory health) suggest a shift toward sustained monitoring and care suitable for non‐professional travelers (Mampre et al. 2025).

A historical mapping review is well suited to clarify not only what has been studied but how “care” has been framed across changing aerospace contexts. Mapping can reveal how certain concerns become recognized as mission‐critical while other domains central to nursing knowledge remain comparatively under‐theorized. Therefore, we conducted a PubMed‐based historical mapping review of aerospace nursing literature indexed between January 1, 1946, and December 30, 2025. By mapping the trajectory across 208 retrieved records (196 included in synthesis), we aimed to identify shifting paradigms of care—from military transport to planetary habitation—and to clarify implications for nursing competencies in the emerging era of commercial spaceflight.

## 2 Methods

### 2.1 Design

We conducted a historical mapping review to trace how aerospace nursing has been framed in the indexed literature across eight decades and to identify major shifts in clinical focus, target populations, and theoretical lenses relevant to habitation beyond Earth. To support transparency in reporting, we summarized the selection process in a PRISMA‐style flow diagram. Phases were developed iteratively through team discussion by aligning recurrent themes with major historical and technological inflection points in aviation and spaceflight. Phase boundaries and labels were refined until we agreed that each phase represented a distinct dominant framing of the aerospace environment and the nursing work foregrounded within it.

### 2.2 Data Sources and Search Strategy

Given the interdisciplinary nature of the topic—combining historical military records with modern physiological research—PubMed (MEDLINE) was selected because of its long‐term coverage of biomedical, nursing, and life sciences literature. PubMed was intentionally used as a single, historically continuous biomedical index to enable conceptual and historical mapping, rather than exhaustive evidence capture across multiple databases.

The search strategy used a combination of Medical Subject Headings and free‐text terms designed to capture the intersection of the aerospace environment and the nursing profession: (“Space Flight”[MeSH] OR “Aerospace Medicine”[MeSH] OR “Microgravity”) AND (“Nursing”[Title/Abstract] OR “Nurse”[Title/Abstract] OR “Nurses”[Title/Abstract]). The search covered January 1, 1946, to December 30, 2025 (last searched December 30, 2025).

### 2.3 Study Selection

Eligibility criteria included peer‐reviewed articles, historical reviews, opinion papers, and theoretical papers that explicitly discussed nursing roles, nursing interventions, or nursing education within aerospace or microgravity environments. Records focused solely on engineering or animal physiology without explicit nursing relevance, such as nursing roles, nursing interventions, nursing education, or clearly articulated nursing‐relevant implications, were excluded. For synthesis, we prioritized records available in English or with an English abstract.

We independently screened titles and abstracts of all retrieved records (*n* = 208). Twelve records were excluded at this stage because of a clear context mismatch or limited relevance to aerospace, spaceflight, or transport nursing (*n* = 12). Full texts were assessed for the remaining records (*n* = 196), and no full‐text records were excluded. Disagreements were resolved through discussion and consensus.

### 2.4 Data Charting and Analysis

Data were extracted using a standardized charting form capturing author(s), year of publication, journal, study type, target population, and primary clinical focus. Data analysis followed a thematic and chronological approach. The literature was categorized into four chronological phases based on historical context and the dominant focus of care, enabling the construction of an evolutionary matrix to visualize paradigm shifts over eight decades. In addition to describing phases chronologically, we compared how each phase framed the environment (transport route, workplace, clinical setting, and living place) and what this implied for the scope of nursing practice and knowledge.

The complete list of records retrieved from PubMed (*n* = 208) is provided in Supporting File 1.

## 3 Results

**Figure 2:**
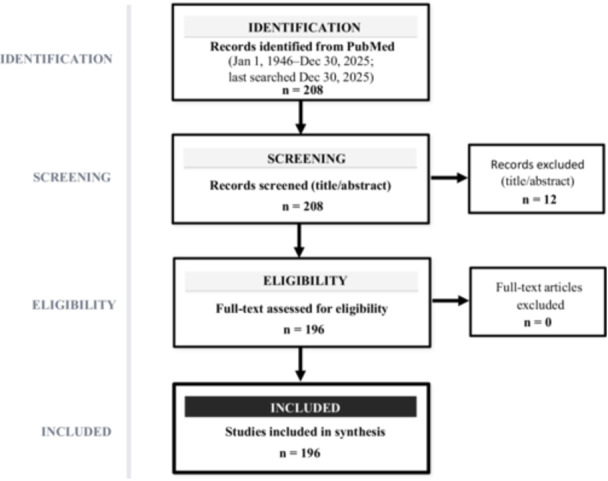
Study selection flow diagram. PubMed retrieval yielded 208 records (January 1, 1946, to December 30, 2025). After title and abstract screening, 12 records were excluded. The remaining 196 records were assessed in full text; no full‐text records were excluded. A total of 196 records were included in the synthesis.

A total of 196 records were included in the synthesis (Figure 2). Chronological and thematic mapping identified four phases in the evolution of aerospace nursing, reflecting changing missions, technologies, and populations. The defining features of each phase are summarized in Table 1. To make the link between historical inflection points, care priorities, and enabling technologies more explicit, Table 1 also summarizes representative vehicles and care platforms associated with each phase, including fixed‐ and rotary‐wing transport, ISS‐based health support, and commercial spaceflight.

**Table 1:** The evolutionary matrix of aerospace nursing (1946–2025).

	Phase 1: The Origins (1946–1970s)	Phase 2: Professionalization (1980s–1990s)	Phase 3: Clinical Adaptation (2000s–2010s)	Phase 4: The New Frontier (2020s–2025)
Historical context	WWII and Vietnam War Jet Age Aviation Development of aeromedical evacuation (AE) systems	Space Shuttle era University‐based nursing education Women entering professional workforce	ISS permanent habitation Standardization of simulation training Technological integration in critical care	Space tourism/commercial spaceflight Artemis/Mars missions Post‐COVID remote medicine
Representative vehicles/care platforms	Fixed‐wing aeromedical evacuation aircraft; early air ambulances	Mature fixed‐wing aeromedical evacuation systems; rotary‐wing critical care transport (e.g., helicopter EMS) in many contexts; Space Shuttle‐era program environments	International Space Station as a sustained platform; analog and simulation environments; telemedicine‐enabled training	Commercial spaceflight (capsule‐based missions); long‐duration analog habitats; planned lunar missions (e.g., Artemis)
Target population	Wounded soldiers (combat casualties, trauma patients)	Flight nurses and astronauts (occupational health of the crew/provider)	Analog patients (simulated patients, mannequins, and special environments)	Civilians and space tourists (diverse health profiles, older adults, and families)
Clinical focus	“Transport and survival” Hypoxia, barotrauma, vibration Focus on getting patient alive to destination	“Occupational health” Stress, fatigue, and circadian rhythms Mental health of the nurse	“Procedure and competency” CPR quality, intubation skills Telemedicine protocols	“Habitation and life support” Chronic disease management Respiratory physiology Disaster response in space
Theoretical lens	Military medicine (triage, logistics, and evacuation chains)	Person–environment nursing theorizing (e.g., Rogers' unitary perspective); occupational health lens	Evidence‐based practice (EBP) (standardization, protocols, and safety training)	Planetary health/holistic care (survival to “living,” bio‐psycho‐social adaptation)
Key literature (representative PMIDs)	Origins and history PMID: 3883982 PMID: 526222—Barger (1979)	Theory and stress PMID: 2235436—Rogers (1990) PMID: 10294706—Whitley et al. (1989)	Training and competency PMID: 28061918—De Jong et al. (2017) PMID: 15555963	New frontier PMID: 40849174—Mampre et al. (2025) PMID: 39319890—Paula et al. (2024)
KEY SHIFT paradigm	Sky as a transport route (passage)	Space as a workplace (professional environment)	Microgravity as a clinical setting (clinical environment)	Space as a living place (habitation)

*Note:* The evolutionary matrix was developed from PubMed records retrieved (*n* = 208); records included in synthesis (*n* = 196). Representative literature (not limited to cited references) is indicated with PMIDs.

### 3.1 Phase 1: The Origins—Sky as a Transport Route (1946–1970s)

The genesis of aerospace nursing is rooted in mid‐20th century military imperatives. Historical accounts situate early flight nursing within wartime evacuation systems and professional formation (Barger 1979). Early nursing descriptions emphasized practical care in air ambulances and evacuation chains under constraint (Graham 1951; Lazaro 1949; Purvis 1947; Thorp 1949). The period also documented operational problems in aero medical nursing and innovations intended to support transport care, including equipment kits and procedural guidance for nursing practice in flight (Thorp 1949; Thorp and Stein 1948). As aviation practice matured, applied writings continued to describe role development and experience‐based knowledge in air evacuation nursing (Alena 1968; Mays 1971). While the Space Age began in the 1960s, early “space age nursing” writings often remained anticipatory, projecting potential nursing roles without clear pathways into routine practice (Barron 1975; Piper and Corrado 1968).

### 3.2 Phase 2: Professionalization—Space as a Workplace (1980s–1990s)

During the Space Shuttle era, the literature shifted from operational survival concerns toward professional identity, provider well‐being, and theoretical foundations. Studies of flight nurses examined occupational stress and job satisfaction and highlighted social support as a buffer against strain, reframing provider well‐being as integral to safe practice (Singh 1990; Whitley et al. 1989). Clinical case discussions also documented circadian disruption and insomnia as occupational issues with practical implications for performance (Amoroso and Bell 1991). In parallel, theoretical work articulated space nursing as a legitimate field and emphasized person–environment relations as a nursing concern in extra‐terrestrial contexts (Malinski 1990; Rogers 1990). Professional and conceptual writings further framed space nursing as a frontier and challenge, reinforcing the need to extend nursing knowledge beyond transport survival tasks (Barrett 1991; Perrin 1985).

### 3.3 Phase 3: Clinical Adaptation—Microgravity as a Clinical Setting (2000s–2010s)

With sustained habitation in low Earth orbit and growing use of simulation, publications increasingly addressed how clinical procedures and training must be adapted to altered gravity and constrained environments. Empirical work evaluated the feasibility and performance of critical interventions during flight, including monitoring approaches to improve in‐flight chest compressions (Thomas et al. 1995). Work on aseptic technique under altered gravity examined how changes in fluid behavior affect sterility and procedural practice, highlighting how microgravity can transform routine infection‐control assumptions (McCuaig 1992). This phase also emphasized competency‐based education and standardization. Studies described the clinical experience and learning styles of flight nurse and aeromedical evacuation technician students and identified clinical education needs through gap analysis, supporting structured preparation for care under constraint (De Jong et al. 2017, 2019). Simulation‐based evaluation of en route care training further underscored the role of standardized scenarios for readiness (DeForest et al. 2018).

### 3.4 Phase 4: The New Frontier—Space as a Living Place (2020s–2025)

Recent literature reflects the shift toward civilian participation, commercial spaceflight, and longer duration missions. Professional updates continued to synthesize role evolution and anticipate future directions for flight and aerospace nursing practice (Shorb 1985). At the same time, long‐standing but underdeveloped concerns about life‐course health re‐emerge as habitation becomes plausible. Work on fetal development and experimental models under altered gravity reinforces that reproduction and development remain scientific frontiers with direct implications for long‐duration habitation (Sekulić et al. 2005; Sekulic et al. 2022). Terrestrial reports of in‐flight births provide an additional analog for rare but high‐stakes reproductive contingencies under constraint (Heggie 2020). Recent nursing perspectives on spaceflight physiology suggest that organ‐specific long‐term management is increasingly being framed as nursing‐relevant, consistent with the shift toward civilian and long‐duration needs (Mampre et al. 2025). Disaster‐competency syntheses further underscore the need for robust preparedness as space traffic increases (Paula et al. 2024).

Across phases, the dominant framing progressively shifted—from the sky as a transport route (passage), to space as a workplace (professional environment), to microgravity as a clinical setting, and most recently to space as a living place (habitation). This reframing is mirrored by changes in the field's center of gravity: from survival‐focused transport care, to attention to provider well‐being and theory, to procedural adaptation and training, and toward habitation‐oriented nursing concerns such as everyday living support, chronic disease management, and life‐course care.

## 4 Discussion

This PubMed‐based historical mapping review traced the 80‐year trajectory of aerospace nursing across 1946–2025 using records retrieved (*n* = 208), with 196 records included in synthesis. The evolutionary matrix highlights a fundamental paradigm shift from aeromedical evacuation—where nursing work is oriented toward survival during transport—to planetary habitation—where nursing work must support living, functioning, and adapting in extra‐terrestrial environments. Across phases, aerospace nursing matured from a sub‐specialty of military medicine into a field with its own occupational concerns, theoretical foundations, and clinical adaptation challenges.

A unifying thread across these phases is the shifting person–environment relationship. In aerospace contexts, the environment is not a backdrop; it actively shapes what counts as risk, comfort, function, dignity, and feasible care. This concern is recognizable across nursing theory traditions and has also been articulated explicitly in space‐nursing scholarship, including Rogers' influential contributions. Using this broad lens allows us to integrate the gaps highlighted in our map—everyday living support, life‐course and reproductive care, chronic illness routines, and workforce and system readiness—under a single question: what becomes mission‐critical nursing knowledge when space is a living place rather than a passage, a workplace, or a clinical setting for discrete procedures?

Beyond documenting topical change, the matrix provides a knowledge‐development narrative: across decades, the field repeatedly renegotiated what counts as “care” as missions, technologies, and populations changed. This reframing is not merely semantic; it shapes what problems are recognized as mission‐relevant, what competencies are legitimized, and what kinds of evidence are produced and circulated. For *Nursing Inquiry*, the implication is that aerospace nursing can be read as a boundary case for nursing knowledge development: it exposes how nursing's objects of concern expand or contract depending on how an environment is framed—as passage, workplace, clinical setting, or living place—and what institutions treat as legitimate “care problems.”

### 4.1 From “Cure” to “Care”: The Necessity of a Holistic Framework

A critical finding of this review is the historical predominance of the medical model—focusing on physiological survival and emergency response—over nursing's holistic model of care. Early aeromedical evacuation accounts operationalized transport care as a distinct nursing practice shaped by flight stressors, logistics, and wartime constraints (Barger 1979; Graham 1951; Lazaro 1949; Purvis 1947; Thorp 1949; Thorp and Stein 1948). Later, provider well‐being became visible as an operational necessity: occupational stress, social support, and sleep disruption were framed as factors affecting safe performance and mission success (Amoroso and Bell 1991; Singh 1990; Whitley et al. 1989). In parallel, as microgravity was increasingly treated as a clinical setting, research emphasized discrete procedures and training performance that could be evaluated and standardized under constraint (DeForest et al. 2018; De Jong et al. 2017, 2019; McCuaig 1992; Thomas et al. 1995). These developments strengthened readiness, yet they also reinforced an orientation toward acute risk, measurable performance, and episodic events.

From a nursing knowledge perspective, these historical framings tend to privilege what can be stabilized, measured, and audited under operational pressure. Over decades, this has helped professionalize aerospace nursing as a specialized practice, but it may also have narrowed the “legitimate” nursing agenda to problems that resemble emergency medicine or procedural performance. The consequence is not simply academic: as the field turns to civilian spaceflight, the everyday conditions of living—privacy, sleep, hygiene, elimination, symptom interpretation, and interpersonal support—become clinically consequential for safety and mission continuity, not optional comforts.

As civilian participation expands, this gap becomes particularly visible. Space tourists and non‐professional travelers will not be trained to tolerate discomfort like astronauts; many will also have diverse functional capacities and chronic comorbidities. In that context, “care” must include maintaining daily functioning and dignity as well as responding to rare emergencies. Operational research on complex mission scheduling underscores that daily care is inseparable from systems design and workflow; what can be done (and when) is shaped by constrained timelines and distributed decision‐making, and health support must be planned accordingly (Marquez et al. 2023). In other words, habitation is as much about the organization of care as it is about the physiology of exposure.

Nursing theory can provide a practical scaffold for reframing these issues by keeping the person–environment relationship central as the meaning of mission‐critical care changes across contexts. Space‐nursing scholarship has historically made this explicit, including Rogers' and Malinski's contributions, and professional calls have long argued that nursing knowledge must extend beyond transport survival toward holistic adaptation and sustained care (Barrett 1991; Perrin 1985; Malinski 1990; Rogers 1990). In this sense, theory is not an embellishment but a way of clarifying what should count as nursing‐relevant evidence as humanity moves from survival to habitation.

### 4.2 Reproduction and the Lifecycle: The Uncharted Frontier

Perhaps the most profound gap identified is the limited and fragmented discourse on reproduction and the female lifecycle in extra‐terrestrial contexts. Work on fetal development under altered gravity highlights the potential importance of gravitational loading for physiological development, raising questions about pregnancy and gestation in altered gravity environments (Sekulić et al. 2005; Sekulic et al. 2022). Although this literature is not primarily nursing‐focused, it creates clear nursing‐relevant implications for counseling, monitoring, and decision‐making in habitation scenarios. Terrestrial aviation reports of in‐flight births also illustrate that rare, high‐stakes reproductive contingencies occur in constrained environments and require rapid coordination, privacy management, and infection control—domains aligned with nursing expertise (Heggie 2020).

If humanity aims for true planetary habitation, the clinical and ethical management of menstruation, contraception, pregnancy, and childbirth must be addressed explicitly. This is a domain where nursing—with its deep expertise in women's health and family‐centered care—should lead agenda setting and ethical analysis. Yet the persistence of silence is likely shaped not only by scientific uncertainty but also by governance and institutional constraints: risk tolerance, liability, and the absence of clearly articulated nursing roles in reproductive contingencies may discourage empirical work and open discussion. Bringing these boundary conditions into view is not merely “adding topics”; it is defining what counts as mission‐critical care in a living place. Habitation without a life‐course perspective is, at best, partial habitation.

### 4.3 Policy and Workforce Implications

The transition to the New Frontier demands a re‐evaluation of workforce preparation. Current competency models are strongly influenced by aeromedical evacuation and en route care traditions, emphasizing stabilization and rare emergencies under constraint (Barger 1979; Graham 1951; Lazaro 1949; Purvis 1947; Thorp 1949; Thorp and Stein 1948; Thomas et al. 1995). At the same time, the literature documents provider well‐being as central to safe practice, suggesting that sustainable workforce models must address stress, sleep, and fatigue as mission‐relevant conditions of work (Amoroso and Bell 1991; Singh 1990; Whitley et al. 1989).

Recent work on disaster competencies underscores that as space traffic increases, systems must be prepared for low‐probability, high‐consequence events and cascading failures (Paula et al. 2024). Evidence from aeromedical education research provides a template for specifying learning needs and using simulation to standardize preparation for constrained environments (DeForest et al. 2018; De Jong et al. 2017, 2019). The next step is to define “minimum viable nursing capability” for commercial spaceflight and long‐duration missions: competencies that integrate procedural readiness with everyday living support, behavioral health, chronic care routines, and team‐based remote decision‐making under constrained resources. Nursing's historical contributions to space programs suggest that these roles will also intersect with systems work—planning, coordination, and operational problem‐solving—alongside direct clinical care (Czerwinski et al. 2000). In practical terms, the evolutionary matrix implies a shift in competency logic: from “can we perform procedures under constraint?” to “can we sustain safe living under constraint?”

### 4.4 Future Research Agenda: Toward Habitation‐Oriented Nursing Knowledge

Building on the evolutionary matrix, we propose a focused research agenda that advances habitation‐oriented nursing knowledge while remaining sensitive to governance, risk tolerance, and feasibility in commercial spaceflight.

1. **Everyday living as mission‐critical care (activities of daily living under altered gravity)**
  A key next step is to treat everyday living as a safety, quality, and ethics domain rather than a comfort afterthought. Early aeromedical evacuation accounts privileged transport survival problems and logistics of care under constraint (Barger 1979; Graham 1951; Lazaro 1949; Purvis 1947; Thorp 1949; Thorp and Stein 1948), while later work privileged procedural performance and standardization (DeForest et al. 2018; De Jong et al. 2017, 2019; McCuaig 1992; Thomas et al. 1995). Habitation‐oriented nursing research should complement these traditions by specifying what safe and dignified daily life requires in altered gravity. Priority questions include the following: What constitutes adequate hygiene, elimination, sleep, nutrition, mobility, and symptom self‐management when body positioning, fluids, and movement patterns change? What nursing assessments are feasible when restraint, limited supplies, and limited privacy are constraints? Which elements of everyday support can be engineered versus what requires nursing presence and relational work? This stream of work should define nursing‐relevant outcomes for habitation (e.g., dignity, functional independence, sleep quality, adherence to routines, and psychological safety) and develop feasible indicators that can be tracked longitudinally across missions and analog environments.
2. **Life‐course, reproductive, and family‐centered care beyond Earth**
  The matrix suggests that reproduction remains a persistent silence despite its centrality to habitation. Available work indicates that gravitational loading may be necessary for normal fetal development, raising questions about pregnancy and gestation in altered gravity environments (Sekulić et al. 2005; Sekulic et al. 2022). Nursing research should translate these concerns into care questions: What monitoring and counseling are required for reproductive decision‐making under uncertainty? What anticipatory guidance should be provided about symptom recognition and escalation when evacuation may be delayed or impossible? Terrestrial analogs such as in‐flight births can inform planning for rare, high‐stakes reproductive contingencies in constrained environments, including privacy management, infection prevention, and coordination under time pressure (Heggie 2020). This domain also requires explicit ethical and governance frameworks for consent, risk communication, and dignity in settings where clinical resources and legal accountability may differ from terrestrial norms.
3. **Habitation with chronic conditions and diverse civilian populations**
  As civilian participation expands, nursing research must assume diversity in age, function, and comorbidity. Organ‐specific nursing perspectives provide an entry point for long‐duration monitoring and care needs (Mampre et al. 2025), but habitation requires integrating chronic disease management with self‐care support and systems‐level triage. Priority questions include how medication routines, symptom monitoring, and adherence support should be adapted when circadian rhythms, fluid distribution, and exertion patterns differ, and how common chronic conditions interact with altered gravity to shape risk for deconditioning, sleep disruption, and anxiety. Operational work on complex spaceflight timelines underscores that caregiving routines must also fit within constrained schedules and decision structures, suggesting a need for nursing‐led workflow and role design studies (Marquez et al. 2023). In practical terms, this line of work should clarify “minimum viable chronic care” in spaceflight—what can be safely supported onboard, what requires remote support, and what constitutes an unacceptable risk for habitation‐oriented missions.
4. **Competency logic and system readiness for commercial spaceflight**
  Workforce research should specify competencies that integrate disaster preparedness with simulation‐informed training and standardization for constrained environments (DeForest et al. 2018; De Jong et al. 2017, 2019; Paula et al. 2024). This includes defining minimum scopes of practice, escalation pathways, and quality and safety indicators for care beyond Earth. The field's historical emphasis on provider well‐being further suggests that competency frameworks should address stress, fatigue, and sleep management as mission‐relevant conditions of practice, rather than individual‐level shortcomings (Amoroso and Bell 1991; Singh 1990; Whitley et al. 1989). Research should also examine how nurses participate in operational planning and coordination as mission systems grow more complex, including how decision authority, documentation, and remote consultation are structured when resource constraints limit conventional escalation models (Marquez et al. 2023).
5. **Theory development for person–environment relations in extra‐terrestrial settings**

Theory is essential not as an abstraction but as a tool for identifying what should count as nursing‐relevant evidence as humanity moves from survival to habitation. Rogers' and Malinski's work provides a starting point for theorizing person–environment relations in space contexts (Malinski 1990; Rogers 1990), complemented by professional calls positioning space nursing as a legitimate field and challenge (Barrett 1991; Perrin 1985). Theory development should proceed alongside empirical work to articulate how nursing concepts of adaptation, well‐being, comfort, dignity, and relational care function when bodily routines and social life are altered. This theoretical work is especially important as spaceflight transitions from professional astronauts to civilians, where expectations of autonomy and quality of life will shape what counts as acceptable care and, ultimately, what “habitation” can mean.

Together, these directions shift the field from a predominantly transport‐ and emergency‐centered orientation toward an explicit account of living, functioning, and caring in extra‐terrestrial environments, and they provide an actionable agenda for nursing knowledge development. Building on this evolutionary matrix, future scholarship could develop middle‐range theory for nursing in extra‐terrestrial and other extreme environments, drawing on person–environment conceptualizations across nursing theory traditions to specify testable concepts and outcomes relevant to habitation.

### 4.5 Limitations

This review used a focused design, which entails several limitations. First, the search was intentionally restricted to PubMed (MEDLINE). While databases such as CINAHL or Embase could capture additional nursing and interdisciplinary sources, PubMed was selected as a historically continuous biomedical index to support conceptual and historical mapping across eight decades in a consistent way. Accordingly, the findings should be interpreted as a PubMed‐indexed map of nursing‐relevant aerospace discourse rather than an exhaustive capture of all relevant literature. Second, synthesis prioritized records available in English or with an English abstract; relevant non‐English work may therefore be under‐represented, particularly given the international nature of space programs and aeromedical services. Third, due to heterogeneity of included publications—ranging from historical narratives and professional commentaries to simulation and observational studies—formal critical appraisal was not undertaken. The goal was not to estimate intervention effects but to trace changes in framing and identify gaps and silences in what has been treated as mission‐critical nursing work. Finally, phase boundaries were interpretive; although phases were developed through reviewer consensus and anchored to major historical and technological inflection points, alternative periodizations are possible. Despite these limitations, the evolutionary matrix provides a transparent, historically grounded framework that can be refined as the field and its evidence base develop.

## 5 Conclusion

Aerospace nursing has evolved from a military necessity to a scientific discipline essential for the future of human spaceflight. The journey from the sky as a transport route to space as a living place highlights the enduring value of nursing's adaptability. As civilian spaceflight expands, the role of the nurse must expand from the guardian of survival to the facilitator of habitation. To achieve this, the discipline must advance nursing knowledge grounded in its theoretical roots, address gaps in everyday living and reproductive care, and actively shape the competencies, systems, and policies that will govern health in the final frontier. In the coming decade, the credibility of “habitation” will hinge not only on engineering reliability but on whether care can be organized to protect dignity, functioning, and well‐being for diverse civilians. Aerospace nursing therefore should not be treated as a downstream operational detail, but as a knowledge‐generating discipline that helps define what safe and livable human presence beyond Earth will require.

## Author Contributions

**Kazumi Kubota:** conceptualization, methodology, investigation, data curation, formal analysis, visualization, writing – original draft, project administration. **Satoko Tsuda:** data curation, formal analysis, writing – review and editing, supervision. **Anna Kubota:** visualization, writing – review and editing, supervision. All authors read and approved the final manuscript.

## Funding

The authors have nothing to report.

## Ethics Statement

Ethical approval was not required because this study analyzed publicly available literature and did not involve human participants, animals, or identifiable private information.

## Conflicts of Interest

The authors declare no conflicts of interest.

## Declaration of Generative AI and AI‐Assisted Technologies in the Writing Process

During the preparation of this work, the authors used ChatGPT (OpenAI) to assist with language editing. After using this tool, the authors reviewed and edited the content as needed and take full responsibility for the content of the publication.

## Supporting information

20251230SupplementaryFile1_FINAL.

## Data Availability

This study used publicly available bibliographic records from PubMed. The search strategy and the complete list of retrieved records (*n* = 208) are provided in Supporting File 1. Data extraction materials are available from the corresponding author upon reasonable request.
