# Supplementary material for: From Aeromedical Evacuation to Planetary Habitation: A PubMed‐Based Historical Mapping Review of Aerospace Nursing (1946–2025)
Source: Nurs Inq. 2026 Mar 30;33(2):e70098. doi: 10.1111/nin.70098 (PMC13035310; doi:10.1111/nin.70098)
Supplement: Supplementary file 1 — 20251230SupplementaryFile1_FINAL. [file NIN-33-e70098-s001.pdf]

Supplementary File 1. A. Complete list of records retrieved from PubMed (n=208)

1. Aebersold, M., Tschannen, D., & Sculli, G. (2013). Improving nursing students' communication skills using crew resource management strategies. *Journal of Nursing Education*, *52*(3), 125–130. https://doi.org/10.3928/01484834-20130205-01
2. Albert, J. (1956). What's different about flight nursing? *American Journal of Nursing*, *56*(7), 873–874.
3. Alena, V. M. (1968). Flight nursing: 1967–improving the present–probing the future–meeting the challenge through applied research. *Aerospace Medicine*, *39*(3), 292–295.
4. Altitude studies for nurses. (1950). *Military Surgeon*, *106*(6), 481–483.
5. Amoroso, P. J., & Bell, N. S. (1991). A nurse's complaint of insomnia. *Aviation, Space, and Environmental Medicine*, *62*(3), 273–274.
6. Barger, J. (1979). Origin of flight nursing in the United States Army Air Forces. *Aviation, Space, and Environmental Medicine*, *50*(11), 1176–1178.
7. Barger, J. (1980). U.S. Army Air Forces Flight Nurses: training and pioneer flight. *Aviation, Space, and Environmental Medicine*, *51*(4), 414–416.
8. Barger, J. (1985a). Flight nurse firsts: the first Flight Nurses Creed. *Aviation, Space, and Environmental Medicine*, *56*(2), 171–172.
9. Barger, J. (1985b). Flight nurse firsts: the first formal flight nurse graduation. *Aviation, Space, and Environmental Medicine*, *56*(3), 275–276.
10. Barger, J. (1985c). Flight nurse firsts: the first flight nurse killed in action. *Aviation, Space, and Environmental Medicine*, *56*(4), 376–377.
11. Barger, J. (1985d). Rivalry for the sky: a prelude to the development of the flight

nurse program in the US Army Air Forces. *Aviation, Space, and Environmental Medicine*, *56*(1), 73–78.

12. Barger, J. (1986). Strategic aeromedical evacuation: the inaugural flight. *Aviation, Space, and Environmental Medicine*, *57*(6), 613–616.
13. Barger, J. (1991a). Coping behaviors of U.S. Army flight nurses in World War II: an oral history. *Aviation, Space, and Environmental Medicine*, *62*(2), 153–157.
14. Barger, J. (1991b). Preparing for war: lessons learned from U.S. Army flight nurses of World War II. *Aviation, Space, and Environmental Medicine*, *62*(8), 772–775.
15. Barrett, E. A. (1991). Space nursing. *Cutis*, *48*(4), 299–303.
16. Barron, N. J. (1975). Development of aerospace nursing. *Aviation, Space, and Environmental Medicine*, *46*(4 Sec 1), 445–446.
17. Baum, A. Z. (1968). Nurse to the astronauts. *RN*, *31*(5), 46–47.
18. Baum, A. Z. (1969). Air Force flight nurses. The world is their ward. *RN*, *32*(10), 36–41.
19. Baum, A. Z. (1971). Busy airport, busy nurses. *RN*, *34*(8), 38–41.
20. Booth, D. (2009). A plastic surgical nurse's military experience. *Plastic Surgical Nursing*, *29*(3), 160–164. https://doi.org/10.1097/PSN.0b013e3181b45b93
21. Boyko, S. M., & Hamilton, K. N. (1994). Know your limitations: the hypoxic flight nurse. *Journal of Emergency Nursing*, *20*(6), 556–558.
22. Braverman, A. (2021). Moral dilemmas of nurses and paramedics during in-flight medical emergencies on commercial airlines. *Journal of Emergency Nursing*, *47*(3), 476–482. https://doi.org/10.1016/j.jen.2020.12.003
23. Brettle, P. (1991). Spaceflight: galactic nursing. *Nursing Standard*, *5*(37), 18–19. https://doi.org/10.7748/ns.5.37.18.s44

24. Bridges, E. J. (2003). Military and disaster nursing. *Critical Care Nursing Clinics of North America*, *15*(2), xiii–xiv. https://doi.org/10.1016/s0899-5885(02)00086-2

25. Bruce, G. L., & Jones, S. A. (1979). Flight Nurse Section–past, present, future. *Aviation, Space, and Environmental Medicine*, *50*(5), 503–507.

26. Burge, J. M. (1992). Living and working in space: evolution of nursing in a new environment. *Holistic Nursing Practice*, *6*(4), 67–74. https://doi.org/10.1097/00004650-199207000-00012

27. Burke, J. B. (1979). The nursing explosion-technology, knowledge, and practice: its implications for leadership in aerospace nursing. *Aviation, Space, and Environmental Medicine*, *50*(4), 405–408.

28. Burke, J. B. (1980). Field interactions in aerovac nursing. *Aviation, Space, and Environmental Medicine*, *51*(6), 618–621.

29. Burke, J. B. (1983). Synergistic management: the task of the flight nurse. *Aviation, Space, and Environmental Medicine*, *54*(3), 261–262.

30. Butcher, H. K., & Forchuk, C. (1992). The overview effect: the impact of space exploration on the evolution of nursing science. *Nursing Science Quarterly*, *5*(3), 118–123. https://doi.org/10.1177/089431849200500308

31. Campbell, P. (1987a). A prototype flight nurse education program. *Journal of Emergency Nursing*, *13*(4), 235–240.

32. Campbell, P. (1987b). Comparison of flight nurses' prehospital assessments and emergency physicians' ED assessments of trauma patients. *Journal of Emergency Nursing*, *13*(4), 219–222.

33. Campbell, P. M. (1987). Flight nurse practice: what is the governing body? *Journal of Emergency Nursing*, *13*(4), 198–199.

34. Chandler, C. J. (1964). The new Air Force nurse. *Nursing Outlook*, *12*, 36–37.

35. Chandler, J. V., & Polk-Walker, G. C. (1989). Health problems in the extraterrestrial environment. *Orthopaedic Nursing*, *8*(5), 51–55, 64. https://doi.org/10.1097/00006416-198909000-00012

36. Chiodo, G. T., Tolle, S. W., & Critchlow, C. (2000). Is there a doctor in the house? *General Dentistry*, *48*(3), 228–232, 234, 236.

37. Chonin, A. (1998). Telehealth: important concepts for future nursing practice in space environments. *Life Support & Biosphere Science*, *5*(4), 433–435.

38. Chonin, A. T. (2004). Pediatric nursing in space environments. *Journal for Specialists in Pediatric Nursing*, *9*(3), 103–105. https://doi.org/10.1111/j.1547-5069.2004.00103.x

39. Christensen, C. (1969). [Conference with American Air Force nurses in Wiesbaden]. *Tidskrift for Sveriges sjukskoterskor*, *36*(11), 665–667.

40. Churchill, J. (2000). Flight nursing. A touch of hope. *Journal of Christian Nursing*, *17*(1), 14–15. https://doi.org/10.1097/00005217-200017010-00006

41. College of Nursing faculty selected to train U.S. Air Force personnel on suicide prevention. (2007). *South Carolina Nurse*, *14*(1), 10.

42. Cook, R. L. (1979). Physician-nurse collaboration: a nurse's perspective. *Aviation, Space, and Environmental Medicine*, *50*(11), 1179–1181.

43. Cook, R. L. (1981). Nursing peer review: a survey of flight nurse section members. *Aviation, Space, and Environmental Medicine*, *52*(1), 50–52.

44. Cowper-Smith, F. (1976). RAF nurses training for in-flight care. *Nursing Mirror and Midwives Journal*, *142*(11), 47–48.

45. Cox, M. K. (1977). Any nurse can prevent dehumanization by the CCU experience.

Aviation, Space, and Environmental Medicine, *48*(6), 568–570.

46. Cruikshank, C. (1995). Space travel, bed rest and nursing. *Nursing Standard*, *10*(11), 52–53. https://doi.org/10.7748/ns.10.11.52.s51
47. Czerwinski, B. S., Plush, L. H., & Bailes, B. K. (2000). Nurses' contributions to the US space program. *AORN Journal*, *71*(5), 1051–1057. https://doi.org/10.1016/s0001-2092(06)61554-8
48. Davies, B. M. (1970). Airport nurse. *Nursing Times*, *66*(44), 1392–1394.
49. Davis, C. (2012). 'Care in the air': the role of in-flight staff. *Emergency Nurse*, *19*(10), 12–15. https://doi.org/10.7748/en2012.03.19.10.12.c8990
50. De Jong, M. J., Dukes, S. F., Dufour, K. M., & Mortimer, D. L. (2017). Clinical experience and learning style of flight nurse and aeromedical evacuation technician students. *Aerospace Medicine and Human Performance*, *88*(1), 23–29. https://doi.org/10.3357/AMHP.4697.2017
51. De Jong, M. J., Dukes, S. F., & Losekamp, T. (2019). Gap analysis to identify clinical education needs of aeromedical evacuation clinicians. *Dimensions of Critical Care Nursing*, *38*(2), 83–89. https://doi.org/10.1097/DCC.0000000000000349
52. De Korne, D. F., Van Wijngaarden, J. D., Van Dyck, C., Hiddema, U. F., & Klazinga, N. S. (2014). Evaluation of aviation-based safety team training in a hospital in The Netherlands. *Journal of Health Organization and Management*, *28*(6), 731–753. https://doi.org/10.1108/jhom-01-2013-0008
53. DeForest, C. A., Blackman, V., Alex, J. E., Reeves, L., Mora, A., Perez, C., Maddry, J., Selby, D., & Walrath, B. (2018). An evaluation of Navy en route care training using a high-fidelity medical simulation scenario of interfacility patient transport. *Military Medicine*, *183*(9-10), e383–e391. https://doi.org/10.1093/milmed/usx129

54. Diamond Jubilee for RAF nurses. (1978). *Nursing Mirror*, *146*(22), 7–9.

55. Donaldson, E., & Pearn, J. (1996). First aid in the air. *Australian and New Zealand Journal of Surgery*, *66*(7), 431–434. https://doi.org/10.1111/j.1445-2197.1996.tb00777.x

56. Dunning, L. (2006). Where nursing skills take flight. *Nursing Times*, *102*(27), 20–21.

57. Dye, B. J. (1975). Occupational health nursing in a military setting. *Occupational Health Nursing*, *23*(2), 12–13. https://doi.org/10.1177/216507997502300202

58. Eastes, L. E. (1989). Evaluating the clinical practice of flight nurses: complexities and innovations. *Journal of Nursing Quality Assurance*, *3*(3), 75–83. https://doi.org/10.1097/00001786-198905000-00009

59. Eaves, R. H., & Flagg, A. J. (2001). The U.S. Air Force pilot simulated medical unit: a teaching strategy with multiple applications. *Journal of Nursing Education*, *40*(3), 110–115. https://doi.org/10.3928/0148-4834-20010301-05

60. Ebbs, N. L., & Timmons, S. (2008). Inter-professional working in the RAF Critical Care Air Support Team (CCAST). *Intensive and Critical Care Nursing*, *24*(1), 51–58. https://doi.org/10.1016/j.iccn.2007.06.003

61. Eby, C. (1971). Nurse is friend to the girls who fly. *Occupational Health Nursing*, *19*(12), 13–16.

62. Edwards, J. (1992). A nursing perspective of Royal Flying Doctor Service. *Australian Nurses Journal*, *21*(6), 23–24.

63. Erler, C. J., Fiege, A. B., & Thompson, C. B. (2000). Flight nurse research activities. *Air Medical Journal*, *19*(1), 13–18. https://doi.org/10.1016/s1067-991x(00)90086-5

64. Famous nurses. Dame Joanna Cruickshank: founder of the RAF Nursing Service. (1979). *Nursing Mirror*, *148*(4), 24.

65. Farrell, B. L., & Allen, M. F. (1973). Physiologic/psychologic changes reported by USAF female flight nurses during flying duties. *Nursing Research*, *22*(1), 31–36.

66. Fouts, B. L., Serres, J. L., Dukes, S. F., Maupin, G. M., Wade, M. E., & Pohlman, D. M. (2015). Investigation of self-reported musculoskeletal injuries on post-deployment health assessment forms for aeromedical evacuation personnel. *Military Medicine*, *180*(12), 1256–1261. https://doi.org/10.7205/MILMED-D-14-00606

67. French, B. C. (1986). After the crash. A school nurse helps Concord students grieve. *Journal of Christian Nursing*, *3*(3), 15–19. https://doi.org/10.1097/00005217-198603030-00005

68. Garrett, J. S. (1998). MedAire: peace of mind in the skies–a flight nurse's dream come true. *Journal of Emergency Nursing*, *24*(1), 71–73. https://doi.org/10.1016/s0099-1767(98)90178-7

69. Gaudinski, M. A. (1979). Coping with expanding nursing practice, knowledge, and technology. *Aviation, Space, and Environmental Medicine*, *50*(10), 1073–1075.

70. Ginsburg, C. M., Henle, G., & Henle, W. (1976). An outbreak of infectious mononucleosis among the personnel of an outpatient clinic. *American Journal of Epidemiology*, *104*(5), 571–575. https://doi.org/10.1093/oxfordjournals.aje.a112332

71. Gisbert Miralles, J., Heintze, C., & Dini, L. (2020). [Delegation modalities for general practitioners in North Rhine-Westphalia: Results of a survey among general practitioners on the assignment of defined tasks to EVA, VERAH and VERAH Plus]. *Zeitschrift für Evidenz, Fortbildung und Qualität im Gesundheitswesen*, *156-157*, 50–58. https://doi.org/10.1016/j.zefq.2020.07.010

72. Golden jubilee of PMRAFNS (Princess Mary's Royal Air Force Nursing Service). (1968). *Nursing Mirror and Midwives Journal*, *127*(3), 32–35.

73. Graham, W. (1951). Flight nursing. *Military Surgeon*, *108*(4), 302–304.

74. Grogan, E. L., Stiles, R. A., France, D. J., Speroff, T., Morris, J. A., Jr, Nixon, B., Gaffney, F. A., Seddon, R., & Pinson, C. W. (2004). The impact of aviation-based teamwork training on the attitudes of health-care professionals. *Journal of the American College of Surgeons*, *199*(6), 843–848. https://doi.org/10.1016/j.jamcollsurg.2004.08.021

75. Guest, J. L. (1989). Nursing frontiers. Critical care in the air. *Journal of Christian Nursing*, *6*(2), 17–21. https://doi.org/10.1097/00005217-198906020-00006

76. Hadbavny, E. A. (1995). All in the life of a flight nurse. *Aviation, Space, and Environmental Medicine*, *66*(3), 284.

77. Harper, P. (2005). Personal experience of aeromedical evacuation of patients in the Balkans. *British Journal of Nursing*, *14*(4), 202–204. https://doi.org/10.12968/bjon.2005.14.4.17603

78. Harper, P. (2006). No pain, no gain: pain behaviour in the armed forces. *British Journal of Nursing*, *15*(10), 548–551. https://doi.org/10.12968/bjon.2006.15.10.21130

79. Hart, M. A. (1980). The evolving role of the flight nurse. *Journal of Emergency Nursing*, *6*(5), 40–41.

80. Hatzfeld, J. J. (2008). Developing evidence-based nursing roles: lessons learned from the health care integrator role. *Nursing Forum*, *43*(2), 72–79. https://doi.org/10.1111/j.1744-6198.2008.00098.x

81. Hayden, G. J. (1958). Nurses and the Air Force Reserve Program. *Military Medicine*, *123*(2), 138–140.

82. Heggie, T. W. (2020). Skyborn: in-flight emergency births on commercial airlines.

Journal of Travel Medicine, *27*(2), taz042. https://doi.org/10.1093/jtm/taz042

83. Hensleigh, C. N. (1987). Flight nursing: opportunities, risks, and rewards. *Journal of Emergency Nursing*, *13*(4), 197.
84. Hibbert, A. (1997). In-flight nursing. *Nursing Standard*, *11*(44), 22–24. https://doi.org/10.7748/ns.11.44.22.s37
85. Hill, M., McLean, M., & Sherwood, E. (1975). Nursing in the sky. *The Canadian Nurse*, *71*(1), 23–26.
86. Hood, S. (2006). Flight nurses are go. *Australian Nursing Journal*, *14*(4), 32.
87. Hoolahan, J. (2004). A pivotal role in international rescue. *Professional Nurse*, *20*(4), 22–23.
88. Howard, P. (1985). Teaching and training in aviation medicine in the United Kingdom. *Journal of UOEH*, *7*(1), 9–25. https://doi.org/10.7888/juoeh.7.9
89. Hsu, N. L. (1973). [Flight nurse]. *Hu Li Za Zhi*, *20*(3), 61–68.
90. Hutchins, J. (1968). Orientation of the aerospace nurse to occupational health. *American Association of Industrial Nurses Journal*, *16*(7), 7–12. https://doi.org/10.1177/216507996801600701
91. Hutchison, B. G., All, A. C., Loving, G. L., & Nishikawa, H. A. (2001). Values identified in different groups of Air Force nurses. *Military Medicine*, *166*(2), 139–145.
92. Isfort, D. A. (1999). So you want to be a flight nurse. *Journal of Emergency Nursing*, *25*(6), 531. https://doi.org/10.1016/s0099-1767(99)70022-x
93. Iveson-Iveson, J. (1981). History of nursing in the Air Force: string, sealing wax and individual daring. *Nursing Mirror*, *153*(3), 27–28.
94. Jackson, L. H. (1989). Memories of a war nurse: Princess Mary's R.A.F. Nursing

Service in Europe. *History of Nursing Bulletin*, *2*(7), 28–36.

95. Janelli, L. M., & Jarmuz, P. A. (1987). Motivational factors that affect the retention of reserve nurses in eight aeromedical evacuation flights. *Aviation, Space, and Environmental Medicine*, *58*(4), 375–378.

96. Joint, T. (2000). Control and prevention of tuberculosis in the United Kingdom: code of practice 2000. Joint Tuberculosis Committee of the British Thoracic Society. *Thorax*, *55*(11), 887–901. https://doi.org/10.1136/thorax.55.11.887

97. Julian, P. W., & Lang, R. A. (1995). Flight nursing: a unique experience. *Aviation, Space, and Environmental Medicine*, *66*(2), 180–181.

98. Kalisch, P. A. (1977). Weavers of scientific patient care: development of nursing research in the U.S. Armed Forces. *Nursing Research*, *26*(4), 253–271.

99. Keirle, O. M. (1961). An airline nursing service. *International Nursing Review*, *8*, 27–33.

100. Kirschke, R. P. (1987). Flight nursing: expect the unexpected. *Journal of Emergency Nursing*, *13*(4), 209–211.

101. La Puma, J., & Balskus, M. (1988). When an indigent patient needs a helicopter: a case report and an accepted institutional policy. *The Journal of Emergency Medicine*, *6*(2), 147–149. https://doi.org/10.1016/0736-4679(88)90159-x

102. Lamb, D. (2003). The introduction of new critical care equipment into the aeromedical evacuation service of the Royal Air Force. *Intensive and Critical Care Nursing*, *19*(2), 92–102. https://doi.org/10.1016/s0964-3397(03)00010-7

103. Latendresse, L. (2004). Medical-surgical nurses flying high. *Medsurg Nursing*, *13*(5), 336–338.

104. Lavernhe, J. P., & Ivanoff, S. (1985). Medical assistance to travellers: a new concept

in insurance–cooperation with an airline. *Aviation, Space, and Environmental Medicine*, *56*(4), 367–370.

105. Lay, F. I. (1953). The flight nurse as a leader. *Military Surgeon*, *113*(3), 196–200.

106. Lazaro, A. R. (1949). The role of the flight nurse in air evacuation. *Military Surgeon*, *105*(1), 60–64.

107. Lee, C. L., Hsiao, Y. C., & Chen, C. Y. (2012). [Roles and functions of military flight nursing: aeromedical evacuation]. *Hu Li Za Zhi*, *59*(3), 23–28.

108. Lee, G. (1987). History of flight nursing. *Journal of Emergency Nursing*, *13*(4), 212–218.

109. Lee, H., Wilbur, J., Kim, M. J., & Miller, A. M. (2008). Psychosocial risk factors for work-related musculoskeletal disorders of the lower-back among long-haul international female flight attendants. *Journal of Advanced Nursing*, *61*(5), 492–502. https://doi.org/10.1111/j.1365-2648.2007.04511.x

110. Leigh Molloy: nursing officer, Air Force. (2011). *Australian Nursing Journal*, *19*(6), 23.

111. Leira, E. C., Lamb, D. L., Nugent, A. S., Ahmed, A., Grimsman, K. J., Clarke, W. R., & Adams, H. P., Jr. (2006). Feasibility of acute clinical trials during aerial interhospital transfer. *Stroke*, *37*(10), 2504–2507. https://doi.org/10.1161/01.STR.0000239661.07675.9d

112. Lenehan, G. P. (1987). A tribute to flight nurses. *Journal of Emergency Nursing*, *13*(4), 191–193.

113. Looper, P. (1987). Inside the interview process for hiring flight nurses: how to survive. *Journal of Emergency Nursing*, *13*(4), 36A–39A.

114. Mace, S. (2000). A nursing career in the RAF. *British Journal of Nursing*, *9*(5), 308.

https://doi.org/10.12968/bjon.2000.9.5.6368

115. Mailey, S. K., & Topley, D. (1999). Flight nursing. *Imprint*, *46*(1), 54–55.

116. Malinski, V. M. (1990). The Rogerian science of unitary human beings as a knowledge base for nursing in space. *National League for Nursing Publications*, *(15-2285)*, 363–374.

117. Mampre, D., Stabenau, K., Thurmond, K., & Petersen, E. (2025). Spaceflight respiratory system from a nursing perspective. *Critical Care Nursing Clinics of North America*, *37*(3), 433–446. https://doi.org/10.1016/j.cnc.2025.05.003

118. Marquez, J. J., Edwards, T., Karasinski, J. A., Lee, C. N., Shyr, M. C., Miller, C. L., & Brandt, S. L. (2023). Human performance of novice schedulers for complex spaceflight operations timelines. *Human Factors*, *65*(6), 1183–1198. https://doi.org/10.1177/00187208211058913

119. Mary, W. (2020). Flight nursing during a pandemic. *American Journal of Nursing*, *120*(7), 13. https://doi.org/10.1097/01.NAJ.0000688128.23416.3b

120. Mays, S. A. (1971). Air evacuation nursing–a personal experience. *AORN Journal*, *14*(5), 79–83. https://doi.org/10.1016/s0001-2092(07)67352-9

121. McCombs, C. M. (1978). Air ambulance services: new horizons for emergency nursing. *Journal of Emergency Nursing*, *4*(4), 21–23.

122. McCuaig, K. (1992). Aseptic technique in microgravity. *Surgery, Gynecology & Obstetrics*, *175*(5), 466–476.

123. McGinnis, P. (1991). The future of occupational health nursing in space. *AAOHN Journal*, *39*(5), 225–230.

124. McNeill, M. M., Pierce, P., Dukes, S., & Bridges, E. J. (2014). En route care patient safety: thoughts from the field. *Military Medicine*, *179*(8 Suppl), 11–18.

https://doi.org/10.7205/MILMED-D-13-00522

125. Mertlich, G., & Quaal, S. J. (1989). Air transport of the patient requiring intra-aortic balloon pumping. *Critical Care Nursing Clinics of North America*, *1*(3), 443–458.

126. Miller, K. H., Sutton, E., & Pantalos, G. (2022). Hierarchical task analysis reimagined as a planning tool for surgery during exploration space flight. *Surgical Innovation*, *29*(5), 616–624. https://doi.org/10.1177/15533506211053210

127. Miller, P. L. (1991). Nursing in space: a new frontier for nursing. *Nursing Management*, *22*(8), 36–37. https://doi.org/10.1097/00006247-199108000-00010

128. Moerschel, T. P. (1987). Air Force nursing has more to offer today than when Cherry Ames was a flight nurse. *Pennsylvania Nurse*, *42*(1), 12–13.

129. Morgan, M. E. (1974). Surgical nurse clinical specialist. *Aerospace Medicine*, *45*(2), 182–184.

130. Morris, P. (1981). RAF nursing today: in sky's the limit. *Nursing Mirror*, *153*(3), 24–26.

131. Neifert, P. L., & Bourgeois, J. A. (2000). Denial of pregnancy: a case study and literature review. *Military Medicine*, *165*(7), 566–568.

132. Nimmo, M. J. (1987). Flight nursing: living it and loving it. *Journal of Emergency Nursing*, *13*(4), 262–263.

133. Novotny, D. R. (1969). Aerospace nursing. *ANA Clinical Conferences*, 76–79.

134. Nugent, W. L., Rhee, K. J., & Wisner, D. H. (1991). Can nurses perform surgical cricothyrotomy with acceptable success and complication rates? *Annals of Emergency Medicine*, *20*(4), 367–370. https://doi.org/10.1016/s0196-0644(05)81656-7

135. Nurses in space. (1974). *Nursing Mirror and Midwives Journal*, *139*(8), 48–50.

136. Nursing at the airport. (1972). *RNABC News*, 12–13.

137. Nursing with the TVA. (1946). *American Journal of Nursing*, *46*, 176–180.

138. O'Connor, V. (1971). Careers in nursing. Nursing link with those men in their flying machines. *Nursing Times*, *67*(45), 1418–1420.

139. Oxer, H. F. (1977). Carriage by air of the seriously ill. *Medical Journal of Australia*, *1*(15), 537–540. https://doi.org/10.5694/j.1326-5377.1977.tb130873.x

140. Paula, B. A. C., Haberland, D. F., Guilherme, F. J. A., Barbosa, B. L., Oliveira, A. B., & Silva, T. A. S. M. D. (2024). Aerospace nurses' competencies in disaster situations: a scoping review. *Revista Latino-Americana de Enfermagem*, *32*, e4326. https://doi.org/10.1590/1518-8345.7421.4326

141. Perrin, M. M. (1985). Space nursing. A professional challenge. *Nursing Clinics of North America*, *20*(3), 497–503.

142. Pierce, P. F. (2005). Monitoring the health of Persian Gulf War veteran women. Federal Nursing Service Award. *Military Medicine*, *170*(5), 349–354. https://doi.org/10.7205/milmed.170.5.349

143. Pierce, P. F., & Evers, K. G. (2003). Global presence: USAF aeromedical evacuation and critical care air transport. *Critical Care Nursing Clinics of North America*, *15*(2), 221–231. https://doi.org/10.1016/s0899-5885(02)00082-5

144. Piper, D. A., & Corrado, V. P. (1968). Space age nursing. *International Nursing Review*, *15*(4), 368–381.

145. Plainte, K. (1987). Profiles: interview with a chief flight nurse. *Journal of Emergency Nursing*, *13*(4), 27A–30A.

146. Plaut, K., Maple, R. L., Wade, C. E., Baer, L. A., & Ronca, A. E. (2003). Effects of hypergravity on mammary metabolic function: gravity acts as a continuum. *Journal*

of Applied Physiology (1985), *95*(6), 2350–2354. https://doi.org/10.1152/japplphysiol.00287.2003

147. Polk-Walker, G. C. (1989). Aerospace nursing: the new frontier. *Journal of Professional Nursing*, *5*(4), 224–230. https://doi.org/10.1016/s8755-7223(89)80055-9

148. Pugh, D. (2000). Flight nursing down under: a perspective. *Accident and Emergency Nursing*, *8*(3), 141–143. https://doi.org/10.1054/aaen.2000.0151

149. Purvis, E. G. (1947a). Nursing care in air ambulances. *American Journal of Nursing*, *47*(3), 158–160. https://doi.org/10.1097/00000446-194703000-00013

150. Purvis, E. G. (1947b). Nursing in air evacuation. *Military Surgeon*, *100*(2), 165–168.

151. Raduenz, S. B. P., Santos, J. L. G. D., Lazzari, D. D., Nascimento, E. R. P. D., Nascimento, K. C. D., & Moreira, A. R. (2020). Nurses' responsibilities in the aerospace environment. *Revista Brasileira de Enfermagem*, *73*(4), e20180777. https://doi.org/10.1590/0034-7167-2018-0777

152. Rappsilber, C. (2007). Chapter 2. Military nursing 1948-1954. *Oklahoma Nurse*, *52*(4), 10–14.

153. Ravella, P. C. (1995). A survey of U.S. Air Force flight nurses' adaptation to service in Vietnam. *Aviation, Space, and Environmental Medicine*, *66*(1), 80–83.

154. Ray, M. A., & Turkel, M. C. (2001). Federal Nursing Service Award. Impact of TriCare/managed care on total force readiness. *Military Medicine*, *166*(4), 281–289.

155. Resnick, S. M., Hall, G. L., Simmer, K. N., Stick, S. M., & Sharp, M. J. (2008). The hypoxia challenge test does not accurately predict hypoxia in flight in ex-preterm neonates. *Chest*, *133*(5), 1161–1166. https://doi.org/10.1378/chest.07-2375

156. Respini, E. M. (1965). Clinical aspects of aerospace nursing. *Aerospace Medicine*,

36, 545–548.

157. Rhoades, S. (2003). Space nursing: expanding the horizons. *Reflections on Nursing Leadership*, *29*(4), 28–30, 44.

158. Ricci, M. A., & Brumsted, J. R. (2012). Crew resource management: using aviation techniques to improve operating room safety. *Aviation, Space, and Environmental Medicine*, *83*(4), 441–444. https://doi.org/10.3357/asem.3149.2012

159. Richards, D. (1975). Nursing seven miles up. *Nursing Mirror and Midwives Journal*, *140*(11), 46–48.

160. Richards, H. (1963). Air escort duty. *Nursing Times*, *59*, 1641–1642.

161. Rivers, F. M., Dukes, S., Hatzfeld, J., Yoder, L. H., Gordon, S., & Simmons, A. (2017). Understanding post-deployment reintegration concerns among en route care nurses: a mixed-methods approach. *Military Medicine*, *182*(S1), 243–250. https://doi.org/10.7205/MILMED-D-16-00209

162. Rodenberg, H. (1992). The revised trauma score: a means to evaluate aeromedical staffing patterns. *Aviation, Space, and Environmental Medicine*, *63*(4), 308–313.

163. Rogers, M. E. (1990a). A conversation with Martha Rogers on nursing in space. *National League for Nursing Publications*, *(15-2285)*, 375–386.

164. Rogers, M. E. (1990b). Nurses in space. *National League for Nursing Publications*, *(41-2281)*, 213–220.

165. Ronca, A. E., & Alberts, J. R. (2000). Physiology of a microgravity environment selected contribution: effects of spaceflight during pregnancy on labor and birth at 1 G. *Journal of Applied Physiology (1985)*, *89*(2), 849–854. https://doi.org/10.1152/jappl.2000.89.2.849

166. Rotondo, G., & Saltarelli, F. (1981). [The Sikorskj HH-3F in rescue activities and in

air medical transport]. *Minerva Medica*, *72*(38), 2535–2548.

167. Rubin, M. (1988). Nursing in space. *Missouri Nurse*, *57*(5), 14.

168. Rudge, F. W. (1995). A review of articles published in Aviation, Space, and Environmental Medicine, 1975-94. *Aviation, Space, and Environmental Medicine*, *66*(10), 1005–1009.

169. Saline, D. (1984). Flight nurse develops health program for Intn'l Airport. *Pennsylvania Nurse*, *39*(9), 5–6.

170. Schiemer, A. (2017). Benign episodic unilateral mydriasis in a flight nurse. *Aerospace Medicine and Human Performance*, *88*(5), 500–502. https://doi.org/10.3357/AMHP.4787.2017

171. Schmelz, J. O., Bridges, E. J., Duong, D. N., & Ley, C. (2003). Care of the critically ill patient in a military unique environment: a program of research. *Critical Care Nursing Clinics of North America*, *15*(2), 171–181. https://doi.org/10.1016/s0899-5885(02)00056-4

172. Schulmeister, L., Fournet, H., & Drew, J. (2001). Is there a nurse on board? *American Journal of Nursing*, *101*(7), 49–52. https://doi.org/10.1097/00000446-200107000-00023

173. Schwartz, R. J., Jacobs, L. M., & Juda, R. J. (1992). Evaluating the compliance of universal precautions by aeromedical personnel before and after an educational seminar on infectious diseases. *Connecticut Medicine*, *56*(1), 3–5.

174. Schweitzer, G., Nascimento, E. R. P. D., Malfussi, L. B. H., Hermida, P. M. V., Nascimento, K. C. D., & Moreira, A. R. (2020). Implementation of the protocol of nursing care in trauma in aeromedical service. *Revista Brasileira de Enfermagem*, *73*(3), e20180516. https://doi.org/10.1590/0034-7167-2018-0516

175. Sekulic, S., Jovanovic, A., Zivanovic, Z., Simic, S., Kesic, S., Petkovic, B., Capo, I., & van Loon, J. J. (2022). Which precocial rodent species is more suitable as the experimental model of microgravity influence on prenatal musculoskeletal development on international space station? *Life Sciences in Space Research*, *33*, 48–57. https://doi.org/10.1016/j.lssr.2022.04.001
176. Sekulić, S. R., Lukac, D. D., & Naumović, N. M. (2005). The fetus cannot exercise like an astronaut: gravity loading is necessary for the physiological development during second half of pregnancy. *Medical Hypotheses*, *64*(2), 221–228. https://doi.org/10.1016/j.mehy.2004.08.012
177. Semonin-Holleran, R. (1994). Flight nursing: providing patient care during air transport. *Accident and Emergency Nursing*, *2*(3), 134–139. https://doi.org/10.1016/0965-2302(94)90159-7
178. Shea, L. E., Adamzczak, P., & Flanagan, T. J. (1987). Transition to flight nursing: three experiences. *Journal of Emergency Nursing*, *13*(4), 31A–35A.
179. Shepherd, L., & Edwards, S. L. (2004). The effects of flying: processes, consequences and prevention. *British Journal of Nursing*, *13*(1), 19–29. https://doi.org/10.12968/bjon.2004.13.Sup4.16347
180. Shorb, G. A. (1985). An update: the flight nurse section–its past, present, and future. *Aviation, Space, and Environmental Medicine*, *56*(5), 466–468.
181. Singh, R. G. (1990). Relationship between occupational stress and social support in flight nurses. *Aviation, Space, and Environmental Medicine*, *61*(4), 349–352.
182. Skinner, R. E. (1983a). The making of the air surgeon: the early life and career of David N. W. Grant. *Aviation, Space, and Environmental Medicine*, *54*(1), 75–82.
183. Skinner, R. E. (1983b). The U.S. Flight Nurse: a supplementary bibliography.

Aviation, Space, and Environmental Medicine, *54*(8), 735–737.

184. Spotlight on transport/flight nursing. (2002). *Nursing*, *32*(7), 78.

185. Sredi, D. R. (1981). Aviation opportunities for occupational health nurses. *Occupational Health Nursing*, *29*(4), 20–21.

186. Stanley, T. (1987). Flight nursing–trend for the future. *Imprint*, *34*(4), 22–24.

187. Stohler, S. A. (2001). High performance team interaction in an air medical program. *Human Performance in Extreme Environments*, *5*(2), 37–40.

188. Stuby, A. L. (1998). [Trip diary: 13 years of service with REGA. A flight nurse reports]. *Krankenpflege. Soins Infirmiers*, *91*(9), 66–68.

189. Symanski, M. E. (2000). A nurse on Mars? Why not? *American Journal of Nursing*, *100*(10), 57–61.

190. Syner, J. C. (1967). Science and technology in space nursing. *AORN Journal*, *5*(1), 71–75. https://doi.org/10.1016/s0001-2092(08)71358-9

191. The past 40 years of nursing in AsMA. (2003). *Aviation, Space, and Environmental Medicine*, *74*(11), 1219.

192. Thomas, F., Clemmer, T. P., & Orme, J. F., Jr. (1985). A survey of advanced trauma life support procedures being performed by physicians and nurses used on hospital aeromedical evacuation services. *Aviation, Space, and Environmental Medicine*, *56*(12), 1213–1215.

193. Thomas, M. M., & Longo, M. R., Jr. (1976). Care of patients after cardiac catheterization. *Aviation, Space, and Environmental Medicine*, *47*(2), 192–198.

194. Thomas, S. H., Stone, C. K., Austin, P. E., March, J. A., & Brinkley, S. (1995). Utilization of a pressure-sensing monitor to improve in-flight chest compressions. *American Journal of Emergency Medicine*, *13*(2), 155–157.

https://doi.org/10.1016/0735-6757(95)90083-7

195. Thorp, F. P. (1949). Problems in aero medical nursing. *Journal of Aviation Medicine*, *20*(2), 136–140.

196. Thorp, F. P., & Stein, M. E. (1948). Development of a flight nurses' utility kit. *Quarterly Research Report*, *60*, 36.

197. Trinosky-Lind, P., & Olson, S. (1998). Students in flight nursing. *Nurse Educator*, *23*(1), 9–11. https://doi.org/10.1097/00006223-199801000-00007

198. Trossman, S. (2004). On the cutting edge. Florida nurses participate in NASA training program. *The American Nurse*, *36*(2), 15.

199. Tucker, R. (1990). Childbearing in space. A theoretical perspective. *Journal of Obstetric, Gynecologic, and Neonatal Nursing*, *19*(4), 344–349. https://doi.org/10.1111/j.1552-6909.1990.tb01656.x

200. Tzou, H. Y. (1972). [Introduction to flight nursing]. *Hu Li Za Zhi*, *19*(1), 50–51.

201. Vuillermin, L. (1999). Flight nurses are the backbone of the Royal Flying Doctor Service and must be prepared for anything. Interview by Dimitri Serghis. *Australian Nursing Journal*, *6*(7), 15.

202. Wagner, H. G., Blasdel, I. C., & Poppen, J. R. (1949). Studies to improve visibility of aircraft by use of suitable exterior paint schemes. *Journal of Aviation Medicine*, *20*(2), 102–113.

203. Weekend flight nurse. (1972). *Occupational Health Nursing*, *20*(5), 11. https://doi.org/10.1177/216507997202000502

204. Whitley, T. W., Benson, N. H., Allison, E. J., Jr, & Revicki, D. A. (1989). Occupational stress and job satisfaction among flight nurses. A survey of National Flight Nurses Association members. *Hospital Aviation*, *8*(7), 6–8, 10–11.

https://doi.org/10.1016/s0740-8315(89)80068-3

205. Yamasaki, M., Shimizu, T., Miyake, M., Miyamoto, Y., Katsuda, S., O-Ishi, H., Nagayama, T., Waki, H., Katahira, K., Wago, H., Okouchi, T., Nagaoka, S., & Mukai, C. (2004). Effects of space flight on the histological characteristics of the aortic depressor nerve in the adult rat: electron microscopic analysis. *Biological Sciences in Space*, *18*(2), 45–51. https://doi.org/10.2187/bss.18.45

206. Yanta, G. M. (1958). The stewardess nurse. *American Journal of Nursing*, *58*(12), 1699. https://doi.org/10.1097/00000446-195812000-00043

207. Yerecke, P. A. (1946). Flight nurses make contribution to industry. *Trained Nurse and Hospital Review*, *116*, 195–199.

208. Zimmermann, P. G. (1998). Aviation nurses form corporation. *Nursing Spectrum (Washington, D.C.)*, *8*(4), 13.
